# Epidermal Growth Factor Receptor Expression in Esophageal Adenocarcinoma: Relationship with Tumor Stage and Survival after Esophagectomy

**DOI:** 10.1155/2012/941954

**Published:** 2012-06-26

**Authors:** Daniel Navarini, Richard R. Gurski, Carlos Augusto Madalosso, Lucas Aita, Luise Meurer, Fernando Fornari

**Affiliations:** ^1^Programa de Pós-Graduação em Ciências Cirúrgicas, Faculdade de Medicina, Universidade Federal do Rio Grande do Sul (UFRGS), 90035003 Porto Alegre, RS, Brazil; ^2^Faculdade de Medicina, Universidade de Passo Fundo, 99010080 Passo Fundo, RS, Brazil; ^3^Serviço de Cirurgia Digestiva, Hospital de Clínicas de Porto Alegre, 90035003 Porto Alegre, RS, Brazil; ^4^Programa de Pós-Graduação: Ciências em Gastroenterologia e Hepatologia, Faculdade de Medicina, UFRGS, 90035003 Porto Alegre, RS, Brazil

## Abstract

*Background and Aims*. Esophageal adenocarcinoma (EA) is an aggressive tumor with increasing incidence in occidental countries. Several prognostic biomarkers have been proposed, including epidermal growth factor receptor (EGFR). The aim of this study was to assess whether EGFR expression predicts EA staging and patient survival. *Methods*. In this historical cohort, consecutive patients with EA managed between 2000 and 2010 were considered eligible for the study. Surgical specimens of patients treated with transhiatal esophagectomy were evaluated to establish EGFR expression and tumor differentiation. Staging was classified according with tumor-node-metastasis (TNM) system. Survival was determined according to either medical register or patient's family contact. *Results*. Thirty-seven patients who underwent esophagectomy without presurgical chemotherapy or radiotherapy were studied. EGFR expression was found in 16 patients (43%). EGFR expression was more frequent as higher was the TNM (I and II = 0% versus III = 47% versus IV = 100%; *P* < 0.001). Average survival in months was significantly shorter in the group of patients with EGFR expression (10.5 versus 21.7; *P* = 0.001). *Conclusions*. In patients with esophageal adenocarcinoma treated with transhiatal esophagectomy, EGFR expression was related to higher TNM staging and shorter survival. EGFR expression might be assumed as a prognostic marker for esophageal adenocarcinoma.

## 1. Introduction

Esophageal adenocarcinoma (EA) is an aggressive tumor with increasing incidence in several countries [1–5]. Optimistic five-year survival reaches 25% in patients treated with esophagectomy [6]. Adverse biological behavior and late diagnosis explain at least in part the poor prognosis of EA [7], pointing to the need for new strategies to improve patient selection and outcome prediction.

Gastroesophageal reflux disease (GERD) is a well-known risk factor for EA, particularly in the presence of Barrett's esophagus. This condition increases the likelihood of EA 30 times [8], with incidence of 1 new case of EA in 200 patients per year [9]. Studies also suggest a higher risk for patients with long-segment Barrett's esophagus and a greater risk in men compared with women [1, 10]. Other established risk factors for EA include obesity and smoking [11–13]. 

Among prognostic tools, tumor staging using TNM system is widely employed in the management of patients with EA [14]. Staging is performed by imaging studies, but in many cases a laparoscopic or thoracoscopic intervention is necessary [15]. Prior studies have introduced biomarkers to predict the prognosis of EA. Mutation in p53 gene was first described as a marker of poor prognosis, regardless of TNM status [16]. More recently, epidermal growth factor receptor (EGFR) has received attention by its prognostic capability giving its participation in the control of epithelial cell multiplication. However, EGFR may be overexpressed in esophageal cancer, either in adenocarcinoma or squamous cell carcinoma [17–19].

 It has been demonstrated that EGFR overexpression may be related with higher pathological TNM (pTNM) staging and poor cellular differentiation in EA patients [18–20]. Furthermore, EGFR has been linked with metastasis and decreased survival in these patients [18–20]. However, such prognostic studies included different surgical approaches for treatment of adenocarcinoma.

The hypothesis of the present study is that EGFR might be a prognostic marker for patients with EA treated with transhiatal esophagectomy, a widely accepted surgical technique [21]. Therefore, the aim of this study was to assess whether EGFR expression predicts tumor staging and survival in EA patients treated with a standardized surgical technique.

## 2. Methods

### 2.1. Patients

In this retrospective cohort, we reviewed all cases of EA managed at Hospital de Clínicas de Porto Alegre (HCPA) between January 2000 and December 2010. Patients were selected if they met the following criteria: (1) adenocarcinoma located in the esophagus or gastroesophageal junction (Siewert I and II); (2) treatment with transhiatal esophagectomy. Patients were excluded according to the following criteria: (1) neoadjuvant treatment with radiotherapy or chemotherapy; and (2) missing of pathology or follow-up data; (3) nonsurgical treatment; (4) Siewert III tumor. Data regarding survival were collected from medical registers or phone contact with patient's family.

This study was conducted according to the rules of the Brazilian Ethics and approved by the Ethical Committee of the HCPA (CONEP 198984/GPPG HCPA 08-300).

### 2.2. Transhiatal Esophagectomy

Patients were operated following a standardized surgical approach carried out by the same surgical team. Transhiatal esophagectomy was performed as described elsewhere [22]. Briefly, patients underwent laparotomy and cervicotomy, followed by diaphragm hiatus opening and esophageal dissection with periesophageal lymphadenectomy. The esophagus was sectioned proximally in the cervical segment and distally combined with proximal gastrectomy. Alimentary transit was reconstructed with anastomosis between gastric tube and cervical esophagus. 

### 2.3. Immunohistochemistry Analysis

Determination of EGFR expression with immunohistochemistry was carried out following a published protocol [23]. Briefly, blocks with tumor tissue were first embedded in paraffin for posterior analysis of slices stained with hematoxylin and eosin. The slices were cut in 5 *μ*m, followed by deparaffinization and rehydration in distilled water. They underwent antigen retrieval with Proteinase K (Dako) for 5 min and washed in distilled water. Subsequently they were immersed in 3% hydrogen peroxide for 15 min to block endogenous peroxidase activity and further washed with distilled water for 5 min. The monoclonal anti-human EGFR, clone H11 (anti-EGFR, Dako) was applied to slices at a dilution of 1 : 200 and incubated for 60 min, rinsed in peroxidase blocking solution (PBS) and incubated with streptavidin (1 : 20 dilution) by 30 min at room temperature, and washed twice with PBS for 5 min. Thereafter, chromogen diaminobenzidine was applied for 5 min, washed in common water for 3 min, and then washed in distilled water. Finally, the slices were stained with hematoxylin for 2 min, dehydrated with alcohol, and mounted for analysis. 

### 2.4. Analysis of EGFR Expression

**Figure 1:**
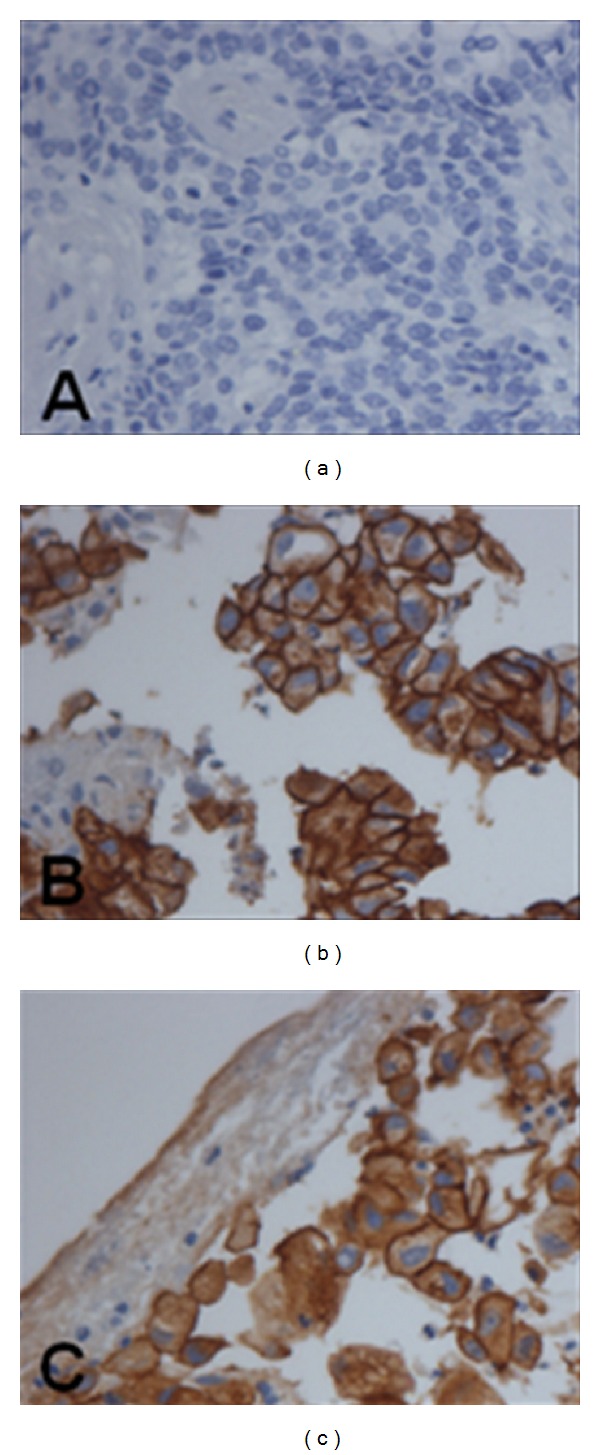
EGFR expression at immunohistochemistry (400X). In (a), a case of adenocarcinoma with negative EGFR. In (b) and (c), 2 different cases of adenocarcinoma with positive EGFR (brown staining).

EGFR expression was considered positive when membrane tumor cell was stained in brown color. An external positive control was performed with placenta tissue and a cell line of esophageal squamous carcinoma with positive EGFR (Figure 1).

Tissue analysis was performed by trained investigators and reviewed by an experienced pathologist blinded to clinical and pathological patient's information.

### 2.5. Statistical Analysis

Data are presented as mean ± SD, and frequencies and percentages when appropriate. The following variables were analyzed: gender, age, tumor place, tumor differentiation, surgical staging, and survival. These variables were related to EGFR expression (yes/no). Quantitative data were analyzed using *t*-test, whereas qualitative variables were tested with chi-square test. Survival was described using Kaplan-Meier analysis. The *P* value was considered statistically significant when ≤0.05.

## 3. Results

A total of 37 patients met the inclusion criteria for the study and had their charts reviewed. Of these, 16 patients (43.2%) had EGFR expression. The characteristics of patients grouped as positive and negative EGFR expression are shown in Table 1. Men represented the majority of patients in both groups. Tumor localization did not differ between groups, with approximately two-thirds located at the GEJ (Siewert I and II), and the remaining in the esophagus. Although well-differentiated tumors were less frequent in EGFR positive patients (44%) as opposed to 76% in EGFR negative, the difference was not statistically significant. Significant differences were found in pTNM staging. EFGR positive tumors presented higher scores either for pT (*T*3 + *T*4 = 94% versus 51%), pN involvement (94% versus 53%), or pM (57% versus 0%), in comparison with EGFR negative lesions. Accordingly tumor staging also differed between groups: all patients with positive EGFR belonged to stages III or IV, whereas most patients (62%) negative for EGFR had stage I or II lesions. EGFR expression was more frequent as higher was the pTNM staging (I and II = 0% versus III = 47% versus IV = 100%; *P* < 0.001).

**Figure 2:**
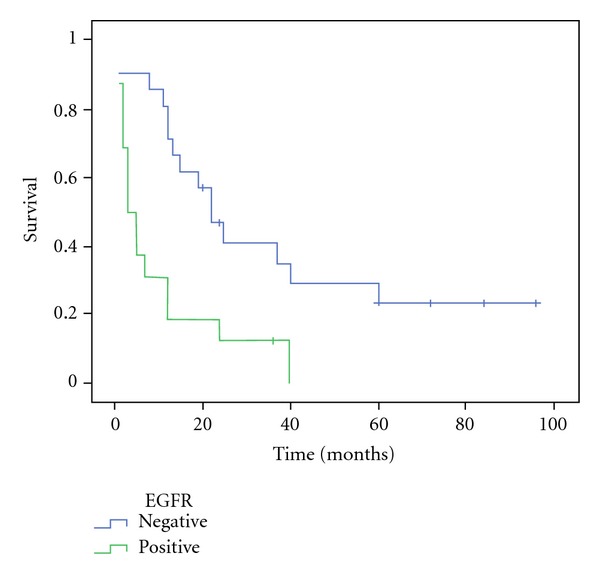
Survival curve (Kaplan-Meier) in patients with and without EGFR expression (4 patients excluded due to surgery-related mortality) (*P* = 0.001).

Out of 37 patients, 4 died soon after the surgery due to operatory complications, including pneumonia and anastomotic leak. As presented in Figure 2, survival was significantly higher in EGFR negative patients compared to those who expressed EGFR (21.7 versus 10.5 months; *P* = 0.001).

## 4. Discussion

Adenocarcinoma of the esophagus and gastroesophageal junction is currently considered a public health problem, given its increasing incidence and poor survival [24]. Efforts to ameliorate outcomes, including optimization of prognostic markers, can be crucial to the management of patients with this condition. Prior studies have suggested that EGFR expression might be useful in predicting outcomes in patients with EA treated with different surgical techniques [18, 19]. The purpose of the present study was to confirm the utility of EGFR expression in the prognosis of patients with this malignant condition treated with a standardized surgical approach characterized by transhiatal esophagectomy. 

The main findings of our study were (1) EGFR expression was related with more advanced lesions, with higher scores for both pTNM classification and tumor staging; (2) There was a trend to the degree of tumor differentiation be poorer in cases with EGFR expression; (3) survival was significantly shorter in the group of patients who expressed EGFR. Secondary findings included a relation between EGFR positivity and older age and predominance of GEJ compromising in spite of esophageal lesions.

In the current study, EGFR expression was found in nearly half of adenocarcinomas. This is in agreement with other studies, in which EGFR expression ranges between 32% and 64% [18, 19, 25, 26]. Besides its relatively high prevalence, EGFR expression was related with more advanced lesions, with higher scores either for tumor staging, nodal involvement, or metastasis. Furthermore, lesions with expressed EGFR showed poorer tumor differentiation. These findings have been demonstrated in other studies [18, 19], indicating that EGFR expression is a marker of more advanced tumors and therefore poorer prognosis.

Survival was significantly shorter in the group of patients who expressed EGFR. This can be explained by several factors, including higher pTNM scores, poorer tumor differentiation and also older age in the group of patients with positive EGFR. These patients showed a trend in receiving more adjuvant treatment with radiochemotherapy after esophagectomy. This likely reflects advanced lesions, which usually require an aggressive approach in spite of surgical treatment [27, 28]. Prior studies have also suggested that EGFR expression is related with shorter survival [18, 19, 25, 26, 29]. It has been proposed that EGFR may participate in the carcinogenesis process of EA [30], based on the fact that EGFR may stimulate proliferation and migration of tumor cells [31, 32]. Further studies are needed to clarify this topic and assess a possible therapeutical benefit of anti-EGFR antibodies [33].

Contrasting with other studies, our patients were treated exclusively with transhiatal esophagectomy before providing tumor specimens for EGFR analysis. Thus, tissue evaluation did not suffer potential influences of other therapeutic modalities, including radiochemotherapy. In addition, EGFR analysis was carried out using immunohistochemistry, which has been considered a feasible technique for this purpose [34]. 

In conclusion, the current study assessed whether EGFR expression predicts tumor staging and survival in EA patients treated with transhiatal esophagectomy. We found that EGFR expression was related with older age, poor tumor differentiation, higher pTNM staging, and shorter survival in comparison with EGFR negative cases. These findings confirm EGFR expression as a prognostic marker in patients with adenocarcinoma of the esophagus and GEJ treated with a standardized surgical approach. Further studies are needed to test the hypothesis that endoscopic assessment of EGFR expression can be useful in the management of EA patients.

## Figures and Tables

**Table 1:** Characteristics of patients with and without EGFR expression.

	EGFR + (*n* = 16)	EGFR – (*n* = 21)	*P*
Age, mean ± SD	70.4 ± 9.0	61.2 ± 7.8	0.002
Men, *n* (%)	13 (81)	18 (86)	0.716
Tumor localization			
Esophageal, *n* (%)	5 (31)	6 (29)	0.999
Siewert I and II	11 (69)	15 (71)	
Tumor differentiation			
Well or moderate	7 (44)	16 (76)	0.086
Poor	9 (56)	5 (24)	
pTNM			
pT1	1 (6)	4 (19)	0.036
2	0	6 (29)	
3	11 (69)	9 (43)	
4	4 (25)	2 (9)	
pN negative	1 (6)	10 (47)	0.010
positive	15 (94)	11 (53)	
pM0	7 ( 43)	21 (100)	<0.0001
1	9 (57)	0	
Tumor staging, *n* (%)			<0.0001
I	0	3 (14)	
II	0	10 (48)	
III	7 (44)	8 (38)	
IV	9 (56)	0	

## References

[1] Bollschweiler E, Wolfgarten E, Gutschow C, Holscher AH (2001). Demographic variations in the rising incidence of esophageal adenocarcinoma in white males. *Cancer*.

[2] Botterweck AAM, Schouten LJ, Volovics A, Dorant E, Van Den Brandt PA (2000). Trends in incidence of adenocarcinoma of the oesophagus and gastric cardia in ten European countries. *International Journal of Epidemiology*.

[3] El-Serag HB (2002). The epidemic of esophageal adenocarcinoma. *Gastroenterology Clinics of North America*.

[4] Kubo A, Corley DA (2002). Marked regional variation in adenocarcinomas of the esophagus and the gastric cardia in the United States. *Cancer*.

[5] Lagarde SM, Ten Kate FJW, Richel DJ, Offerhaus GJA, Van Lanschot JJB (2007). Molecular prognostic factors in adenocarcinoma of the esophagus and gastroesophageal junction. *Annals of Surgical Oncology*.

[6] Wu PC, Posner MC (2003). The role of surgery in the management of oesophageal cancer. *The Lancet Oncology*.

[7] Gertler R, Stein HJ, Langer R (2011). Long-term outcome of 2920 patients with cancers of the esophagus and esophagogastric junction: evaluation of the new union internationale contre le cancer/American joint cancer committee staging system. *Annals of Surgery*.

[8] Solaymani-Dodaran M, Logan RFA, West J, Card T, Coupland C (2004). Risk of oesophageal cancer in Barrett’s oesophagus and gastro-oesophageal reflux. *Gut*.

[9] Yousef F, Cardwell C, Cantwell MM, Galway K, Johnston BT, Murray L (2008). The incidence of esophageal cancer and high-grade dysplasia in Barrett’s esophagus: a systematic review and meta-analysis. *American Journal of Epidemiology*.

[10] Rudolph RE, Vaughan TL, Storer BE (2000). Effect of segment length on risk for neoplastic progression in patients with Barrett esophagus. *Annals of Internal Medicine*.

[11] Brown LM, Swanson CA, Gridley G (1995). Adenocarcinoma of the esophagus: role of obesity and diet. *Journal of the National Cancer Institute*.

[12] Gammon MD, Schoenberg JB, Ahsan H (1997). Tobacco, alcohol, and socioeconomic status and adenocarcinomas of the esophagus and gastric cardia. *Journal of the National Cancer Institute*.

[13] Lagergren J, Bergström R, Nyrén O (1999). Association between body mass and adenocarcinoma of the esophagus and gastric cardia. *Annals of Internal Medicine*.

[14] Edge SB, Compton CC (2010). The american joint committee on cancer: the 7th edition of the AJCC cancer staging manual and the future of TNM. *Annals of Surgical Oncology*.

[15] DeMeester SR (2006). Adenocarcinoma of the esophagus and cardia: a review of the disease and its treatment. *Annals of Surgical Oncology*.

[16] Ireland AP, Shibata DK, Chandrasoma P, Lord RVN, Peters JH, DeMeester TR (2000). Clinical significance of p53 mutations in adenocarcinoma of the esophagus and cardia. *Annals of Surgery*.

[17] Gibault L, Metges JP, Conan-Charlet V (2005). Diffuse EGFR staining is associated with reduced overall survival in locally advanced oesophageal squamous cell cancer. *British Journal of Cancer*.

[18] Wang KL, Wu TT, Choi IS (2007). Expression of epidermal growth factor receptor in esophageal and esophagogastric junction adenocarcinomas: association with poor outcome. *Cancer*.

[19] Wilkinson NW, Black JD, Roukhadze E (2004). Epidermal growth factor receptor expression correlates with histologic grade in resected esophageal adenocarcinoma. *Journal of Gastrointestinal Surgery*.

[20] Langer R, Von Rahden BHA, Nahrig J (2006). Prognostic significance of expression patterns of c-erbB-2, p53, p16 INK4A, p27KIP1, cyclin D1 and epidermal growth factor receptor in oesophageal adenocarcinoma: a tissue microarray study. *Journal of Clinical Pathology*.

[21] Orringer MB, Marshall B, Iannettoni MD (1999). Transhiatal esophagectomy: clinical experience and refinements. *Annals of Surgery*.

[22] Orringer MB, Sloan H (1978). Esophagectomy without thoracotomy. *Journal of Thoracic and Cardiovascular Surgery*.

[23] Hidalgo M, Siu LL, Nemunaitis J (2001). Phase I and pharmacologic study of OSI-774, an epidermal growth factor receptor tyrosine kinase inhibitor, in patients with advanced solid malignancies. *Journal of Clinical Oncology*.

[24] Pera M, Manterola C, Vidal O, Grande L (2005). Epidemiology of esophageal adenocarcinoma. *Journal of Surgical Oncology*.

[25] Al-Kasspooles M, Moore JH, Orringer MB, Beer DG (1993). Amplification and over-expression of the EGFR and erbB-2 genes in human esophageal adenocarcinomas. *International Journal of Cancer*.

[26] Yacoub L, Goldman H, Odze RD (1997). Transforming growth factor-*α*, epidermal growth factor receptor, and MiB-1 expression in Barrett’s-associated neoplasia: correlation with prognosis. *Modern Pathology*.

[27] Bedard EL, Inculet RI, Malthaner RA, Brecevic E, Vincent M, Dar R (2001). The role of surgery and postoperative chemoradiation therapy in patients with lymph node positive esophageal carcinoma. *Cancer*.

[28] Rice TW, Adelstein DJ, Chidel MA (2003). Benefit of postoperative adjuvant chemoradiotherapy in locoregionally advanced esophageal carcinoma. *Journal of Thoracic and Cardiovascular Surgery*.

[29] Gibson MK, Abraham SC, Wu TT (2003). Epidermal growth factor receptor, p53 mutation, and pathological response predict survival in patients with locally advanced esophageal cancer treated with preoperative chemoradiotherapy. *Clinical Cancer Research*.

[30] Poller DN, Steele RJC, Morrell K (1992). Epidermal growth factor receptor expression in Barrett’s esophagus. *Archives of Pathology and Laboratory Medicine*.

[31] Baselga J (2002). Why the epidermal growth factor receptor? The rationale for cancer therapy. *Oncologist*.

[32] Tedesco KL, Lockhart AC, Berlin JD (2004). The epidermal growth factor receptor as a target for gastrointestinal cancer therapy. *Current Treatment Options in Oncology*.

[33] Zhu Z (2007). Targeted cancer therapies based on antibodies directed against epidermal growth factor receptor: status and perspectives. *Acta Pharmacologica Sinica*.

[34] Arteaga CL (2002). Epidermal growth factor receptor dependence in human tumors: more than just expression?. *Oncologist*.

